# Analytical Modelling and Optimization of a Piezoelectric Cantilever Energy Harvester with In-Span Attachment

**DOI:** 10.3390/mi11060591

**Published:** 2020-06-13

**Authors:** Abbas Homayouni-Amlashi, Abdenbi Mohand-Ousaid, Micky Rakotondrabe

**Affiliations:** 1 Laboratoire Génie de Production, National School of Engineering in Tarbes (ENIT), Toulouse INP, University of Toulouse, 47, Avenue d’Azereix, 65000 Tarbes, France; abbas.homayouni@femto-st.fr; 2 Department of Automatic Control and Micro-Mechatronic Systems, FEMTO-ST Institute, Université Bourgogne Franche-Comté, CNRS, 24 rue Alain Savary, 25000 Besançon, France; abdenbi.mohand@femto-st.fr

**Keywords:** energy harvesting, cantilever configuration, piezoelectric patch, neural network, genetic algorithm

## Abstract

In this paper, the location of masses and of a piezoelectric patch for energy harvesting reported onto a vibrating cantilever beam is studied and optimized. To this aim, a genetic algorithm is adapted and utilized to optimize the voltage amplitude generated by the piezoelectric patches by choosing attachment mass, attachment mass moment of inertia, attachment location, piezoelectric patch location and force location on the beam as parameters. While an analytical approach is proposed to evaluate the voltage amplitude, a multi-layer perceptron neural network is trained by the derived characteristic matrix to obtain an approximate function for natural frequencies based on the attachment parameters. The trained network is then used in the core of genetic algorithm to find the best optimization variables for any excitation frequency. Numerical simulation by COMSOL Multiphysics finite element software validates the calculated voltage by analytical approach. The optimization method successfully matches the natural frequency of the beam with the excitation frequency which therefore maximizes the output energy. On the other hand, the superiority of the optimized design over the conventional configuration in harvesting the energy at high frequency excitation is also approved.

## 1. Introduction

Energy harvesting by piezoelectric materials has attracted lots of interests during the last years due to its high power density and architectural simplicity [1]. Piezoelectric materials are generally used in the vibration based energy harvesting specially at small scales [2]. Among different types of structures which have been developed to scavenge the energy from the ambient vibrations sources [3,4,5,6,7], the conventional and still widely used configuration in this case is cantilever based piezoelectric energy harvester which is deeply investigated in several researches by Erturk et al. [8,9,10,11]. In order to maximize the harvested energy from a piezoelectric cantilever beam, previous works considered geometry and parameters optimization on the piezoelectric cantilever beam [12,13,14,15,16] by using topology optimization [17] or by using interval techniques [18], or added a tip attachment as a proof mass to match the fundamental natural frequency of the beam to the excitation frequency [19,20]. However, in some applications with high excitation frequencies like automotive engines, industrial machinery or micro dimension applications the excitation frequency can exceed the fundamental natural frequency of the beam. Then, the best location for the attachment to have maximum piezoelectric voltage may not be the tip of the beam due to vibrational mode shapes. For this case, Erturk et al. [21] investigated the higher modal energy harvesting with a unimorph beam without attachment while the possibility of having the attachment in-span of the beam can be considered. On the other hand, studies on energy harvesting from high frequency excitation with having the mass in-span of the cantilever beam received a restricted attention. Researches in this area did not consider modelling [22] or the piezoelectric electromechanical coupling effect [23]. Furthermore, in all of the researches mentioned above, attached mass on the beam is modelled as a lumped mass and the effects of mass moment of inertia on the harvested energy are not investigated.

In piezoelectric cantilever configuration, it is usual to have piezoelectric layers which cover the whole beam from clamped side to free end [9,10,24,25,26]. This configuration is mostly convenient for the microscale dimensions. However, in the mesoscale dimensions this configuration suffers from low power density since the most strain occurs close to the clamped side [4]. Besides, in excitation frequency close to higher vibrational modes, charge cancellation may happens and continuous electrodes should be avoided [21]. Because of these reasons some researches prefer to have a piezoelectric patch mounted to a passive (non-piezoelectric) beam with the length of the piezoelectric patch being smaller than that of the beam [4,19,27,28]. Generally, in these cases, the piezoelectric patch are mounted near to clamped side of the beam to take the advantage of the maximum strain. However, for excitation frequencies higher than the first natural frequency, again the clamped side may not be the best place for the piezoelectric patch.

**Figure 1:**
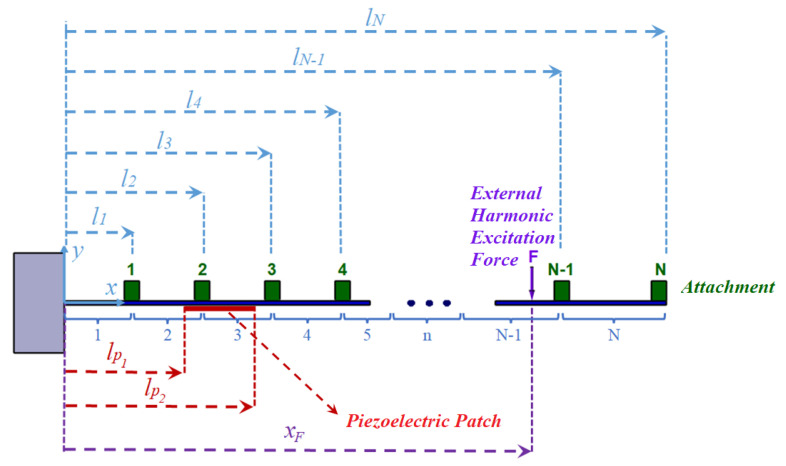
Cantilever beam with several in-span attachments under external harmonic excitation.

In this paper, for a general case of a given passive cantilever beam with mounted piezoelectric patch and several in-span attachments as shown in Figure 1, the voltage amplitude of the piezoelectric patch is found by analytical approach by extending the existing vibrational analysis in the literature [29,30,31,32]. In this vibrational analysis, the method of sectioning the beam between the attachments are utilized to find the response of the beam due to excitation. As such, the model proposed for the voltage amplitude is general for any number of attachment. On the other hand, in order to optimize the harvested energy from an external source of vibration, optimization is applied on a particular case of a beam with one in-span attachment. The derived equation for piezoelectric voltage is used as objective function of the optimization algorithm. In this case, the employed optimization variables are: attachment mass, attachment mass moment of inertia, attachment location, piezoelectric patch location and external force location. In order to deal with this multi parameter optimization, genetic algorithm (GA) is used. However the attachment parameters do not appear in the voltage equation while affecting the natural frequencies. To handle this, one can use the Rayleigh’s quotient method to calculate the fundamental natural frequency of a cantilever beam with one in-span attachment analytically [33]. But, for higher natural frequencies the Rayleigh’s quotient method is almost impossible to be used since there is no information about the shape functions of the beam for higher modes and using polynomial function as shape function [34] can produce significant error especially with in-span attachment on the beam. Therefore, here a two steps optimization is suggested.

First, a multi-layer perceptron (MLP) neural network is trained in order to obtain approximate functions for the natural frequencies based on the attachment parameters. Second, the trained network is used in the evaluation process of the genetic algorithm to form a neural network based genetic algorithm which can find the best combination of the optimization variables to match one of the natural frequencies of the beam to the excitation frequency and maximize the piezoelectric voltage. Both GA and MLP are already explored in the literature. However, utilization of MLP in the evaluation process of the GA has two advantages: first, it can increase the speed of GA algorithm significantly which is a known bottleneck of the GA algorithm in the literature. The second advantage is that MLP can have very high accuracy in calculation of the natural frequencies if the training data is sufficient. These advantages will be explained in details in the text.

To validate the analytical modelling and proposed optimization method of this paper, commercial finite element software COMSOL Multiphysics (COMSOL, Inc., Burlington, NJ, USA) is used. The performance of optimization method in matching the natural frequency of the structure with the excitation frequency is also assessed.

The rest of the paper is organized as follows—in Section 2, vibrational analysis is performed and the piezoelectric voltage due to external excitation is found. In Section 3, first, neural network fitting approach and genetic algorithm are described separately, then their combination in maximizing the voltage amplitude based on the excitation frequency is presented. Section 4 is devoted to simulation results in which the piezoelectric voltage from the proposed approach is compared with conventional method in the literature. Finally, conclusions are discussed in Section 5.

## 2. Modelling

For a beam with several in-span attachments and a piezoelectric patch which is mounted on the beam as shown in Figure 1, the regular method for deriving the equation of motion in transverse vibration is to separate the beam between the attachments. In this case, the equations of motion for each section by considering electrical coupling is in the following form [9]:

(1) $$\begin{array}{c} EI\frac{\partial^{4}w_{n}\left(x,t\right)}{\partial x^{4}}+\mu \frac{\partial^{2}w_{n}\left(x,t\right)}{\partial t^{2}}+C\frac{\partial w_{n}\left(x,t\right)}{\partial t}+\vartheta v\left(t\right)\left[\frac{d\delta (x-l_{p1})}{dx}-\frac{d\delta (x-l_{p2})}{dx}\right]=F\left(t\right)\delta \left(x-x_{F}\right). \end{array}$$

In (1), $w_{n}\left(x,t\right)$ is the transverse displacement, *n* is the number of each segment, EI is the flexural rigidity of the beam, μ is the mass per unit length, *C* is the damping constant, F(t) is the external force, $x_{F}$ is the application location of the external force and δ is the Dirac delta function. The coupling term ϑ which comes from the electrical circuit can be written as [9],

(2) $$\begin{array}{c} \vartheta =-\frac{EI_{p}d_{31}b_{p}}{2h_{p}}\left(h_{c}^{2}-h_{b}^{2}\right), \end{array}$$

where, $EI_{p}$ is the flexural rigidity of the piezoelectric patch, $d_{31}$ is the piezoelectric coupling coefficient, $h_{p}$ and $b_{p}$ are piezoelectric thickness and width respectively while $h_{b}$ is the beam thickness. $h_{c}$ is the distance from the top of piezoelectric layer to the neutral axis. It should be noted that the term $\left[\frac{d\delta (x-l_{p1})}{dx}-\frac{d\delta (x-l_{p2})}{dx}\right]$ specifies the end points of the piezoelectric patch on the beam; and for the sections of the beam where there is no piezoelectric patch, this term is equal to zero. This term is due to the fact that induced voltage in the piezoelectric patch generates point moments at its boundaries which affects the mechanical response of the beam.

Now, to find the piezoelectric voltage due to external mechanical excitation, the response of partial differential Equation (1) should be found. It is worthwhile to mention that the thickness and length of the piezoelectric patch is much less than the beam. As such the rigidity of the piezoelectric patch is negligible in comparison to the beam. In addition, the effects of the attachment’s geometry on the beam’s structural dynamic are highly superior to the effects of the piezoelectric patch geometry. Therefore, in the following modal analysis of the beam, the geometry of the piezoelectric patch is neglected in comparison to the beam and attachment.

### 2.1. Free Vibration Analysis

By following the Ansatz separation, solution of (1) is expressed in the following form:

(3) $$\begin{array}{c} w_{n}\left(x,t\right)=\varphi_{n}\left(x\right)q\left(t\right). \end{array}$$

$\varphi_{n}\left(x\right)$ is the mode shape of the beam and q(t) is the generalized time-dependent coordinate which satisfies

(4) $$\begin{array}{c} \frac{d^{2}q\left(t\right)}{dt^{2}}=-\omega^{2}q\left(t\right). \end{array}$$

In (4), ω is the natural frequency of the system. In order to find the natural frequencies and mode shapes of the beam with several in-span attachments, the external force and damping constant in (1) are considered to be zero. As such, the equations of motion for each segment of the beam in short circuit condition (v(t)=0) is converted to the following eigenvalue problem [35,36]:

(5) $$\begin{array}{c} EI\frac{\partial^{4}\varphi_{n}\left(x\right)}{\partial x^{4}}-\omega^{2}\mu \varphi_{n}\left(x\right)=0. \end{array}$$

Now based on (5), the exact solution for the eigenfunctions which are the mode shapes of the beam, is in the following form [35,36]:

(6) $$\begin{array}{c} \varphi_{n}\left(x\right)=A_{n}\sin\beta x+B_{n}\cos\beta x+C_{n}\sinh\beta x+D_{n}\cosh\beta x, \end{array}$$

in which,

(7) $$\begin{array}{c} \beta_{}^{4}=\mu \frac{\omega^{2}}{EI}. \end{array}$$

In (6), $A_{n}$, $B_{n}$, $C_{n}$ and $D_{n}$ are unknown constants that can be found by applying proper boundary and continuity equations. The clamped end boundary condition is written in the following form:

(8) $$\begin{array}{c} \varphi_{1}\left(0\right)=\varphi_{1}^{\prime }\left(0\right)=0. \end{array}$$

For the attachment point of each segment, the continuity of deformations and equilibrium of forces and moments can be written as

(9) $$\begin{array}{c} \varphi_{n}\left(l_{n}\right)-\varphi_{n+1}\left(l_{n+1}\right)=0\\ {\varphi^{\prime }}_{n}\left(l_{n}\right)-{\varphi^{\prime }}_{n+1}\left(l_{n+1}\right)=0\\ {\varphi^{\prime \prime }}_{n}\left(l_{n}\right)-{\varphi^{\prime \prime }}_{n+1}\left(l_{n}\right)-\frac{\beta^{4}}{\mu }J_{n}{\varphi^{\prime }}_{n}\left(l_{n}\right)=0\\ {\varphi^{\prime \prime \prime }}_{n}\left(l_{n}\right)-{\varphi^{\prime \prime \prime }}_{n+1}\left(l_{n}\right)+\frac{\beta^{4}}{\mu }M_{n}\varphi_{n}\left(l_{n}\right)=0. \end{array}$$

$M_{n}$ is the mass of the attachment and $J_{n}$ is the related moment of inertia of the attachment. Finally, for the free end with attachment, boundary conditions are

(10) $$\begin{array}{c} {\varphi^{\prime \prime }}_{N}\left(L\right)-\frac{\beta^{4}}{\mu }J_{N}{\varphi^{\prime }}_{N}\left(L\right)=0\\ {\varphi^{\prime \prime \prime }}_{N}\left(L\right)+\frac{\beta^{4}}{\mu }M_{N}\varphi_{N}\left(L\right)=0. \end{array}$$

By applying (8)–(10) to (6), the following characteristic equation is formed:

(11) $$\begin{array}{c} {\bar{J}}_{4N\times 4N}P_{4N\times 1}=0, \end{array}$$

where ${\bar{J}}_{4N\times 4N}$ is the characteristic matrix which is the function of β. On the other hand, β is a function of ω based on the explicit expression mentioned in (7). In (11), $P_{4N\times 1}$ is the matrix of the mode shapes constants,

(12) $$\begin{array}{c} P={\left[A_{1},B_{1},C_{1},D_{1},\cdots ,A_{N},B_{N},C_{N},D_{N}\right]}_{1\times 4N}^{T}. \end{array}$$

To find the nontrivial solution of (11), the determinant of the characteristic matrix ${\bar{J}}_{4N\times 4N}$ should be zero which forms the frequency equation and numerical approach should be used to find the natural frequencies. On the other hand, by setting the determinant of characteristic matrix to zero, characteristic Equation (11) will become undetermined. In this case, by integrating the normalization condition, the set of equations become solvable and the coefficients in vector $P_{4N\times 1}$ are found. In this method, orthogonality condition can be used to find the perfect normalization. The generalized orthogonality condition which results in a decoupled ODE of motion for a beam with in-span attachments is expressed in the following form [31,32]:

(13) $$\begin{array}{c} \sum_{n=1}^{N}\int_{l_{n-1}}^{l_{n}}\mu \left(\varphi_{n}^{\left(r\right)}\left(x\right)\varphi_{n}^{\left(s\right)}\left(x\right)\right)dx+M_{n}\varphi_{n}^{\left(r\right)}\left(l_{n}\right)\varphi_{n}^{\left(s\right)}\left(l_{n}\right)+J_{n}{\varphi_{n}^{\prime }}^{\left(r\right)}\left(l_{n}\right){\varphi_{n}^{\prime }}^{\left(s\right)}\left(l_{n}\right)=0\\ (r\neq s). \end{array}$$

This orthogonality condition has a companion form as,

(14) $$\begin{array}{c} \sum_{n=1}^{N}EI\int_{l_{n-1}}^{l_{n}}{\varphi_{n}^{\prime \prime }}^{\left(r\right)}\left(x\right){\varphi_{n}^{\prime \prime }}^{\left(s\right)}\left(x\right)dx=0\;(r\neq s). \end{array}$$

In (13) and (14), (r) and (s) are the numbers of mode. Now, based on (13), the normalization condition can be written for the case when (r=s) and in this case the right hand side of (13) is equal to 1. After integrating the normalization condition to the set of (8)–(10), the coefficients in vector $P_{4N\times 1}$ in (12) are found and the mode shapes can be found for each natural frequencies. Then, the displacement for each point of the beam with considering the beam segments can be found by using the expansion theorem in the following form:

(15) $$\begin{array}{c} w_{n}\left(x,t\right)=\sum_{r=1}^{\infty }\varphi_{n}^{\left(r\right)}\left(x\right)q^{\left(r\right)}\left(t\right). \end{array}$$

### 2.2. Forced Vibration Analysis

By substituting (15) in (1) and multiplying each side of the equation by $\varphi_{n}^{\left(s\right)}$ and integrating over the length of the section, the following equation is derived:

(16) $$\begin{array}{c} \sum_{r=1}^{\infty }q^{\left(r\right)}\left(t\right)\int_{l_{n-1}}^{l_{n}}EI\varphi_{n}^{\left(s\right)}\left(x\right)\frac{\partial^{4}\varphi_{n}^{\left(r\right)}\left(x\right)}{\partial x^{4}}dx+\sum_{r=1}^{\infty }{\ddot{q}}^{\left(r\right)}\int_{l_{n-1}}^{l_{n}}\mu \varphi_{n}^{\left(s\right)}\left(x\right)\varphi_{n}^{\left(r\right)}\left(x\right)\left(t\right)dx+\\ \sum_{r=1}^{\infty }{\dot{q}}^{\left(r\right)}\int_{l_{n-1}}^{l_{n}}C\varphi_{n}^{\left(s\right)}\left(x\right)\varphi_{n}^{\left(r\right)}\left(x\right)\left(t\right)dx+\int_{l_{n-1}}^{l_{n}}\varphi_{n}^{\left(s\right)}\vartheta v\left(t\right)\left[\frac{d\delta (x-l_{p1})}{dx}-\frac{d\delta (x-l_{p2})}{dx}\right]dx=\\ \int_{l_{n-1}}^{l_{n}}\varphi_{n}^{\left(s\right)}\left(x\right)F\left(t\right)\delta \left(x-x_{F}\right)dx. \end{array}$$

Equation (16) is true for each section of the beam. By summing these equations for all sections in the entire length of the beam, the following familiar form of ordinary differential equation (ODE) of motion is derived:

(17) $$\begin{array}{c} \bar{M}\ddot{\Delta }+\bar{C}\dot{\Delta }+\bar{K}\Delta =\bar{F}-\bar{V}, \end{array}$$

in which,

(18) $$\begin{array}{c} {\bar{M}}_{rs}=\int_{0}^{L}\mu \varphi^{\left(s\right)}\left(x\right)\varphi^{\left(r\right)}\left(x\right)\left(t\right)+\sum_{n=1}^{N}M_{n}\varphi^{\left(s\right)}\left(l_{n}\right)\varphi^{\left(r\right)}\left(l_{n}\right)+\sum_{n=1}^{N}J_{n}{\varphi^{\prime }}^{\left(s\right)}\left(l_{n}\right){\varphi^{\prime }}^{\left(r\right)}\left(l_{n}\right) \end{array}$$

(19) $$\begin{array}{c} {\bar{C}}_{rs}=\int_{0}^{L}C\varphi^{\left(s\right)}\left(x\right)\varphi^{\left(r\right)}\left(x\right)\left(t\right) \end{array}$$

(20) $$\begin{array}{c} {\bar{K}}_{rs}=\int_{0}^{L}EI{\varphi^{\prime \prime }}^{\left(s\right)}\left(x\right){\varphi^{\prime \prime }}^{\left(r\right)}\left(x\right)dx \end{array}$$

(21) $$\begin{array}{c} {\bar{F}}_{r}=F\left(t\right)\varphi^{\left(r\right)}\left(x_{f}\right) \end{array}$$

(22) $$\begin{array}{c} \bar{V}=\vartheta v\left(t\right)\left(\varphi^{\prime (r)}\left(l_{p2}\right)-\varphi^{\prime (r)}\left(l_{p1}\right)\right) \end{array}$$

(23) $$\begin{array}{c} \Delta ={\left[\begin{array}{cccc} q^{\left(1\right)} & q^{\left(2\right)} & \cdots & q^{\left(r\right)} \end{array}\right]}^{T} \end{array}$$

(24) $$\begin{array}{c} \varphi^{\left(r\right)}\left(x\right)=\sum_{n=1}^{N}\varphi_{n}^{\left(r\right)}\left(x\right)\left[H\left(x-l_{n-1}\right)-H\left(x-l_{n}\right)\right]. \end{array}$$

In (17), ${\bar{M}}_{rs}$ is the element of the mass matrix, ${\bar{C}}_{rs}$ is the element of the damping matrix, ${\bar{K}}_{rs}$ is the element of the stiffness matrix, ${\bar{F}}_{r}$ is the modal vector’s element of external forces, $\bar{V}$ is the vector of the electromechanical coupling voltage, Δ is the vector of the time dependent coordinates and *H* is the Heaviside function. Now, by applying the orthogonality conditions and normalization in (13) and (14), the mass and stiffness matrices can be rewritten in the following form:

(25) $$\begin{array}{c} {\bar{M}}_{rs}=\delta_{rs}\;{\bar{K}}_{rs}={\left(\omega^{\left(r\right)}\right)}^{2}\delta_{rs}, \end{array}$$

where $\delta_{rs}$ is the Kronecker delta symbol. Based on (25), mass and stiffness matrices are diagonal while the damping matrix is not. For solving the equation of motion in (17) analytically, it is better to have a set of decoupled ODE which is impossible with non-diagonal damping matrix. However, the damping of a system is a model parameter and it is not a physical parameter [31]. Therefore, it is possible to assume a modal damping matrix for the system in the following form:

(26) $$\begin{array}{c} C_{rs}=2\zeta^{\left(r\right)}\omega^{\left(r\right)}\delta_{rs}, \end{array}$$

where $\zeta^{\left(r\right)}$ is the modal damping constant. It is possible to define the numerical value for this modal damping constant by using the Rayleigh damping theory which is common in the Finite Element Method (FEM) software. The Rayleigh damping can be defined as

(27) $$\begin{array}{c} C_{rs}=\alpha {\bar{M}}_{rs}+\beta {\bar{K}}_{rs}, \end{array}$$

where α, β are the Rayleigh’s damping coefficients. By substituting (25) and (26) in to (27), the modal damping can be calculated as

(28) $$\begin{array}{c} \zeta^{\left(r\right)}=\frac{\alpha +\beta {\left(\omega^{\left(r\right)}\right)}^{2}}{2\omega^{\left(r\right)}}. \end{array}$$

Now, by considering diagonal damping matrix, the decoupled set of ODE of motion can be written in the following form:

(29) $$\begin{array}{c} {\ddot{q}}^{\left(r\right)}\left(t\right)+2\zeta^{\left(r\right)}\omega^{\left(r\right)}{\dot{q}}^{\left(r\right)}+{\left(\omega^{\left(r\right)}\right)}^{2}q^{\left(r\right)}\left(t\right)=F\left(t\right)\varphi^{\left(r\right)}\left(x_{f}\right)-\vartheta v\left(t\right)\left(\varphi^{\prime (r)}\left(l_{p2}\right)-\varphi^{\prime (r)}\left(l_{p1}\right)\right), \end{array}$$

v(t) in (29) is found by the electrical circuit equation with mechanical coupling as follows [9]:

(30) $$\begin{array}{c} \dot{v}\left(t\right)+\frac{h_{p}}{R_{l}\varepsilon_{33}^{s}bL_{p}}v\left(t\right)=\sum_{r=1}^{\infty }\varphi^{\left(r\right)}{\dot{q}}^{\left(r\right)}\left(t\right) \end{array}$$

and

(31) $$\begin{array}{c} \varphi^{\left(r\right)}=-\frac{d_{31}EI_{p}h_{pc}h_{p}}{\varepsilon_{33}^{S}L_{p}}\int_{lp1}^{l_{p2}}\frac{d^{2}\varphi^{\left(r\right)}\left(x\right)}{dx^{2}}dx=-\frac{d_{31}EI_{p}h_{pc}h_{p}}{\varepsilon_{33}^{S}L_{p}}\left(\varphi^{\prime (r)}\left(l_{p2}\right)-\varphi^{\prime (r)}\left(l_{p1}\right)\right). \end{array}$$

In (30), $R_{l}$ is the resistive load, $L_{p}$ is the piezoelectric length, $\varepsilon_{33}^{S}$ is the piezoelectric permittivity constant at constant strain by assuming plan-stress assumptions and $h_{pc}$ is the distance of the piezoelectric patch center in thickness direction to the neutral axis. In order to solve (30) and (29) first, external harmonic force is modelled by $F\left(t\right)=F_{0}e^{j\Omega t}$, where Ω is the excitation frequency. By considering a linear electromechanical system, the response of v(t) is also harmonic in terms of $v\left(t\right)=V_{0}e^{j\Omega t}$ while $V_{0}$ and $F_{0}$ are the amplitudes of the voltage and external force respectively. Now, the particular solution of non-homogeneous differential equation in (29) is:

(32) $$\begin{array}{c} q^{\left(r\right)}\left(t\right)=\frac{\left[\varphi^{\left(r\right)}\left(x_{F}\right)F_{0}-V_{0}\vartheta \left(\varphi^{\prime (r)}\left(l_{p2}\right)-\varphi^{\prime (r)}\left(l_{p1}\right)\right)\right]e^{j\Omega t}}{{\left(\omega^{\left(r\right)}\right)}^{2}-\Omega^{2}+j2\zeta_{r}\omega^{\left(r\right)}\Omega }. \end{array}$$

By substituting (32) in (30) the following equation for voltage amplitude is derived:

(33) $$\begin{array}{c} V_{0}=\frac{\sum_{r=1}^{\infty }\frac{j\Omega \Phi^{\left(r\right)}\varphi^{\left(r\right)}\left(x_{F}\right)F_{0}}{{\left(\omega^{\left(r\right)}\right)}^{2}-\Omega^{2}+j2\zeta^{\left(r\right)}\omega^{\left(r\right)}\Omega }}{\sum_{r=1}^{\infty }\frac{j\Omega \Phi^{\left(r\right)}\vartheta \left(\varphi^{\prime (r)}\left(l_{p2}\right)-\varphi^{\prime (r)}\left(l_{p1}\right)\right)}{{\left(\omega^{\left(r\right)}\right)}^{2}-\Omega^{2}+j2\zeta^{\left(r\right)}\omega^{\left(r\right)}\Omega }+\frac{1+j\Omega \tau_{c}}{\tau_{c}}}, \end{array}$$

in which,

(34) $$\begin{array}{c} \tau_{c}=\frac{R_{l}\varepsilon_{33}^{S}bL_{p}}{h_{p}} \end{array}$$

$\tau_{c}$ is the time constant of the electrical circuit. The voltage equation in (33) is similar to one which is reported by Erturk et al. [9]. However, the difference lies on calculating the natural frequencies ($\omega^{\left(r\right)}$) and mode shapes ($\varphi^{\left(r\right)}$) which are based on modal analysis of a beam with in-span attachments. After finding the amplitude of voltage, the displacement of each point of the beam with the help of (15) and (32) can be found by the following equation:

(35) $$\begin{array}{c} w\left(x,t\right)=\sum_{r=1}^{\infty }\varphi^{\left(r\right)}\left(x\right)\times \frac{\left[\varphi^{\left(r\right)}\left(x_{F}\right)F-V_{0}\vartheta \left(\varphi^{\prime (r)}\left(l_{p2}\right)-\varphi^{\prime (r)}\left(l_{p1}\right)\right)\right]e^{j\Omega t}}{{\left(\omega^{\left(r\right)}\right)}^{2}-\Omega^{2}+j2\zeta_{r}\omega^{\left(r\right)}\Omega }. \end{array}$$

As can be seen in (33), voltage amplitude is a complex number which has an absolute value and a phase angle. In the framework of this paper, just the absolute value is important for optimization.

## 3. Optimization

It is desired to have the maximum possible of piezoelectric voltage amplitude in (33) for any excitation frequency. The geometrical optimization on the beam and piezoelectric has been studied before [12,37]. Therefore, by considering constant geometrical parameters for beam and piezoelectric patch and considering just one attachment on the beam, the optimization variables that can influence the voltage amplitude in (33) are piezoelectric patch location ($l_{p1}$), force location ($x_{F}$), attachment location ($l_{1}$), attachment mass (*M*) and attachment mass moment of inertia (*J*). Among these optimization variables, force location and piezoelectric patch location directly appear in the voltage amplitude (33). However, the other three optimization variables related to attachment do not directly appear in the voltage amplitude. Instead, they affect the continuity conditions in (9), and only these latter affect the characteristic matrix in (11) which itself affects the natural frequencies and mode shapes that appear in the voltage amplitude (33). On the other hand, there is no closed form expression between the natural frequencies and the attachment parameters. In fact, there is just closed form expression for first (fundamental) natural frequency of the beam with in-span attachment based on the Rayleigh’s quotient method [33]. But, for higher natural frequencies the Rayleigh’s quotient method cannot be used since there is no information about the shape functions without knowing the natural frequencies. As such, performing optimization based on the attachment parameters is a challenge that will be addressed in the next section.

### 3.1. Multi Layer Perceptron Neural Network

In this section, neural network fitting algorithm in MATLAB software is utilized for the purpose of finding approximate functions for the natural frequencies based on the optimization variables. The algorithm is based on the multi-layer perceptron (MLP) which is a class of artificial neural network. MLP consists three layers of artificial neurons—input layer, output layer and hidden layers. There is just one input layer and one output layer in the MLP network and the number of neurons in input and output layers are equal to the number of input and output variables. However, there can be different numbers of hidden layers in MLP and the main method to increase the performance of the MLP is to define the adequate number of hidden layers which can be done with the trial and error approach.

Having this background, it is now desired to have a MLP network that can get attachment parameters and gives the natural frequencies. To do so, the MLP network should be trained with the training data which consist of input data and target data. Input data are optimization variables regarding the attachment including attachment location, attachment mass and attachment mass moment of inertia. Target data consist of natural frequencies related to the input data. In this paper maximum number of natural frequencies and mode shapes for modal analysis is considered to be 4 which is sufficient enough to model a cantilever even under high excitation frequencies. Therefore, each set of input data consists of 3 optimization variables, which has a set of target data consist of 4 natural frequencies.

The MLP network should have the ability to get any combination of the attachment parameters and give the related natural frequencies. To form the training data for this MLP network, 20 different attachment masses in the domain of (0<M<μL), 20 different attachment mass moments of inertia in the domain of ($0<J<0.0042\;\mu L^{3}$) and 20 different attachment locations in the domain of ($0<l_{1}<L$) have been chosen to form 8000 combinations of attachment parameters. For each of these combinations, there are 4 natural frequencies which should be found by the numerical approach on the characteristic matrix in (11). Therefore, input training data is a matrix with 3 rows and 8000 columns while the target data is a matrix with 4 rows and 8000 columns. Number of hidden layers is 50 and the Bayesian Regularization method has been used to train the network.

**Figure 2:**
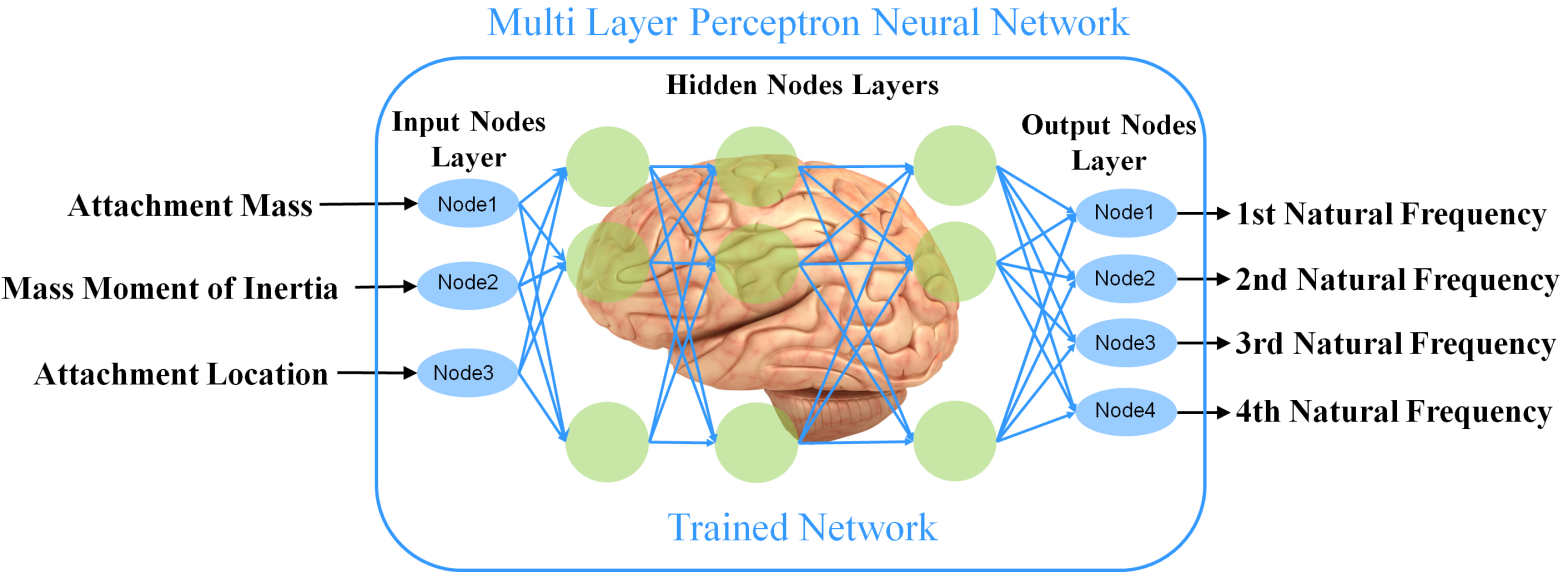
Multi layer perceptron neural network.

After successful training of the network by obtaining natural frequencies for finite set of variables, a continuous function is approximated as a black box which can get any variable in the predefined domain and gives the related natural frequencies immediately as shown in Figure 2 and it is not necessary to calculate the natural frequencies by numerical approach on the characteristic matrix in Equation (11). This will boost the speed in the evaluation procedure of the GA optimization which will be discussed later.

### 3.2. Finding Analytical Expression for Mode Shape Constants

By using MLP neural network, the natural frequencies in (33) are found as functions of the attachment parameters. But, in voltage amplitude (33), mode shapes are also functions of the attachment parameters since attachment parameters affect the continuity (9) and mode shapes constants in (12). Finding analytical expressions for the mode shape constants based on (8)–(10) are almost impossible due to huge size of the expression for each of the constants. Alternative approach proposed by Naguleswaran [36,38] is used here to decrease the number of the unknown constants in (12).

**Figure 3:**
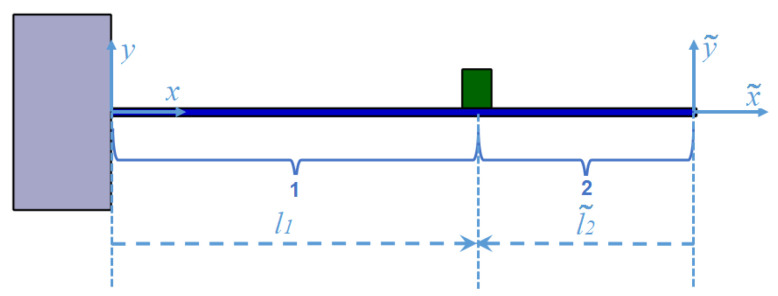
Cantilever beam with two different coordinate system.

For the case when the beam has just one in-span attachment, some algebraic simplification can be done on the mode shapes by defining two coordinate systems for two sections of the beam as shown in Figure 3. The reference of the second coordinate system has been placed at the tip of the beam and the points in the second coordinate system are shown by “ $\tilde{}$”.

The following equation is true for the relation between the points in the first and the points in the second coordinate:

(36) $$\begin{array}{c} \tilde{x}=x-L \end{array}$$

By applying the clamped end boundary conditions mentioned in (8) to Section 1 of the beam, the following result related to the mode shape constant of the first section is obtained:

(37) $$\begin{array}{c} -A_{1}=C_{1}\;-B_{1}=D_{1}. \end{array}$$

Now, by considering the second coordinate system for the second section of the beam and considering no tip mass for the beam, the free boundary condition can be written for the second section of the beam in the following form:

(38) $$\begin{array}{c} \frac{d^{2}\varphi_{2}\left(\tilde{0}\right)}{d{\tilde{x}}^{2}}=\frac{d^{3}\varphi_{2}\left(\tilde{0}\right)}{d{\tilde{x}}^{3}}=0. \end{array}$$

By applying (38) to the mode shape of the second section of the beam, (39) is obtained:

(39) $$\begin{array}{c} A_{2}=C_{2}\;B_{2}=D_{2}. \end{array}$$

Using (37) and (39), mode shapes for Segment 1 and 2 can be written as

(40) $$\begin{array}{c} \varphi_{1}\left(x\right)=A_{1}\left(\sin\beta x-\sinh\beta x\right)+B_{1}\left(\cos\beta x-\cosh\beta x\right)\\ \varphi_{2}\left(\tilde{x}\right)=A_{2}\left(\sin\beta \tilde{x}+\sinh\beta \tilde{x}\right)+B_{2}\left(\cos\beta \tilde{x}+\cosh\beta \tilde{x}\right). \end{array}$$

By defining two coordinate systems, there are 4 unknown constants instead of 8 unknown constant. These 4 unknown constants can be found by rewriting (9) for the new coordinate system,

(41) $$\begin{array}{c} \varphi_{1}\left(l_{1}\right)-\varphi_{2}\left(\tilde{l_{2}}\right)=0\\ {\varphi^{\prime }}_{1}\left(l_{1}\right)-\varphi_{2}^{\prime }\left(\tilde{l_{2}}\right)=0\\ {\varphi^{\prime \prime }}_{1}\left(l_{1}\right)-{\varphi^{\prime \prime }}_{2}\left(\tilde{l_{2}}\right)-\frac{\beta^{4}}{\mu }J_{1}{\varphi^{\prime }}_{1}\left(l_{1}\right)=0\\ {\varphi^{\prime \prime \prime }}_{1}\left(l_{1}\right)-{\varphi^{\prime \prime \prime }}_{2}\left(\tilde{l_{2}}\right)+\frac{\beta^{4}}{\mu }M_{1}\varphi_{1}\left(l_{1}\right)=0. \end{array}$$

Now, to find each constant, first $B_{2}$ is considered to be 1. Then, the first three conditions of (41) are solved analytically with the help of MAPLE software to find the remaining three constants as functions of the natural frequencies and attachment parameters. The code which is written in MAPLE software is mentioned in the Appendix A. On the other hand, in this case there is no normalization in the mode shapes and the mass matrix in (17) is not an identity matrix while it is still diagonal. Therefore, (29) without normalization will be in this form:

(42) $$\begin{array}{c} {\bar{M}}_{rr}{\ddot{q}}^{\left(r\right)}\left(t\right)+{\bar{M}}_{rr}2\zeta^{\left(r\right)}\omega^{\left(r\right)}{\dot{q}}^{\left(r\right)}+{\bar{M}}_{rr}{\left(\omega^{\left(r\right)}\right)}^{2}q^{\left(r\right)}\left(t\right)=\\ F\left(t\right)\varphi^{\left(r\right)}\left(x_{f}\right)-\vartheta v\left(t\right)\left({\varphi_{n}^{\prime }}^{\left(r\right)}\left(l_{p2}\right)-{\varphi_{n}^{\prime }}^{\left(r\right)}\left(l_{p1}\right)\right). \end{array}$$

By considering (42) instead of (29), the mass matrix ${\bar{M}}_{rr}$ enters the piezoelectric voltage amplitude (33) and this latter is rewritten in the following form:

(43) $$\begin{array}{c} V_{0}=\frac{\sum_{r=1}^{\infty }\frac{1}{{\bar{M}}_{rr}}\frac{j\Omega \Phi^{\left(r\right)}\varphi^{\left(r\right)}\left(x_{F}\right)F_{0}}{{\left(\omega^{\left(r\right)}\right)}^{2}-\Omega^{2}+j2\zeta^{\left(r\right)}\omega^{\left(r\right)}\Omega }}{\sum_{r=1}^{\infty }\frac{1}{{\bar{M}}_{rr}}\frac{j\Omega \Phi^{\left(r\right)}\vartheta \left(\varphi^{\prime (r)}\left(l_{p2}\right)-\varphi^{\prime (r)}\left(l_{p1}\right)\right)}{{\left(\omega^{\left(r\right)}\right)}^{2}-\Omega^{2}+j2\zeta^{\left(r\right)}\omega^{\left(r\right)}\Omega }+\frac{1+j\Omega \tau_{c}}{\tau_{c}}}. \end{array}$$

The value of ${\bar{M}}_{rr}$ is found analytically as function of the mode shape constants and attachment specifications by Maple software and it is reported in Appendix A.

In the approach of the last two sections, natural frequencies and mode shapes have been found as functions of the attachment specifications.

### 3.3. Genetic Algorithm Optimization

Now, it is desired to find the optimal voltage amplitude from (43) by finding the optimal position of the attachment, the attachment mass, mass moment of inertia, external force location, and piezoelectric patch location. As such, genetic algorithm is utilized in this paper, which has a great performance in dealing with multi variable optimization problems. Genetic algorithm is an optimization method that works based on the evolution theory and it is completely a simulation of real life in nature. By choosing the voltage amplitude (43) as fitness function to be optimized, the mathematical framework of GA optimization method works in the following steps:

- A number of individuals is chosen which forms the first generation of the population. Each individual is a possible solution for the optimization of the fitness function. Higher number of individuals decreases the possibility of being trapped in the local optimums. here, 200 individuals are considered in the population.
- Pairs of individuals are selected as parents with Roulette wheel method. The ratio of individuals selected as parents to the overall individuals is 0.8.
- Two selected parents give birth to two offspring (two new possible solution) in the crossover procedure.
- Then chromosomes of some offspring will be changed in mutation procedures. The chromosomes for the optimization problem are the optimization variables. The percentage of offspring who experienced the mutation to the whole population is called mutation percentage which is considered here to be 10 percent.
- The last step is the evaluation procedure in which individuals with lowest fitness value will be replaced by offspring. These newly born offspring with remaining individuals form the next generation. To calculate the fitness value of each individual, MLP network and analytical expression for the mode shape constants should be used which will be explained in the next section.
- Newly formed generation will undergo the same procedure of the previous generation.

MATLAB GA toolbox performs the aforementioned steps and stops after a restricted number of times.

### 3.4. Neural Network Based Genetic Algorithm (NN-GA)

**Figure 4:**
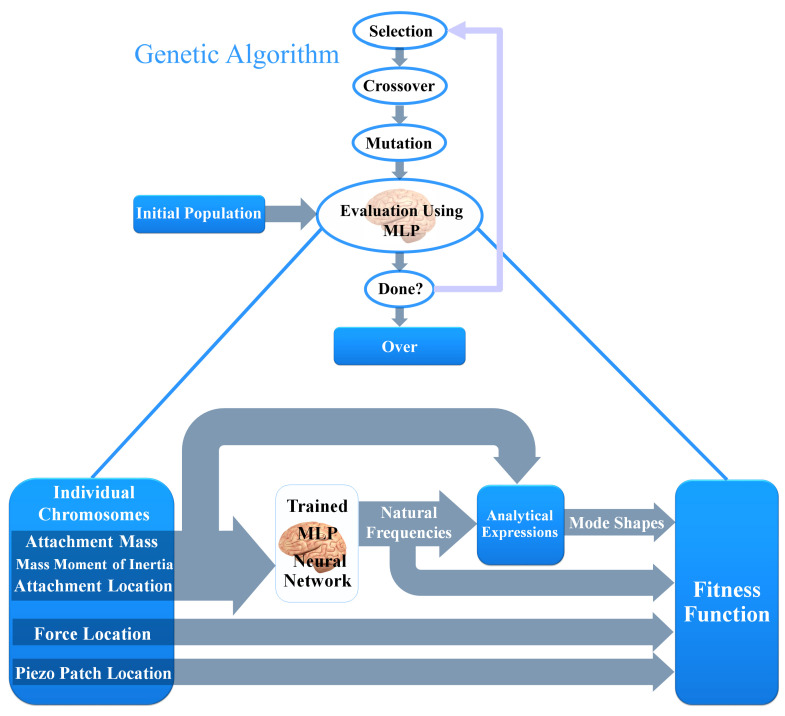
Diagram of neural network based genetic algorithm (NN-GA).

In the evaluation step of genetic algorithm, the fitness value of each individual should be determined. As has been shown in Figure 4, each individual has 5 chromosomes in which 3 of them are related to the attachment. MLP neural network calculates the natural frequencies of the system based on the attachment parameters. Then, mode shapes are calculated by the analytical expressions for the mode shape constants. Finally, with the help of mode shapes, natural frequencies, force location and piezoelectric patch location, the fitness value of each individual are calculated by using the voltage amplitude (43).

Using MLP neural network in the evaluation process of GA will increase optimization speed significantly by eliminating the cumbersome numerical procedures to find the natural frequencies related to each individual. Therefore, it is possible to choose the GA parameters (population number, mutation percentage, ...) different to those mentioned above to compare the results and to avoid trapping in the local optimums.

## 4. Simulation and Results

In this section, first the effect of each optimization variables on the voltage amplitude is investigated solely. It should be noted that the effects of the electrical circuit parameters on the voltage is not investigated here. Therefore, for constant electrical circuit parameters particularly the resistance, the optimization of voltage is equivalent to optimization of power. Investigations are performed on a cantilever beam with an in-span attachment which has a cubic shape with length ($L_{M}$), thickness ($H_{M}$) and width ($W_{M}$). All the features and dimensions of the system are reported in Table 1. In next part of results, the neural network based genetic algorithm is used to find the best optimization variables based on the excitation frequency of the external force. The natural frequencies of the optimized structures are calculated to asses the performance of the optimization method in matching one of the natural frequencies of the structure with the excitation frequency. Then, by simulating the optimized structure in the COMSOL FEM platform, the natural frequencies and voltages which are derived by analytical method are compared with ones obtained by FEM simulation in COMSOL. To investigate the performance of the optimization method, the voltages of the optimized structures are also compared with the classical configuration in which a lumped mass is attached at the tip of the beam and a piezoelectric patch mounted on clamped side of the beam and the results are discussed at the end.

### 4.1. Discussion

**Figure 5:**
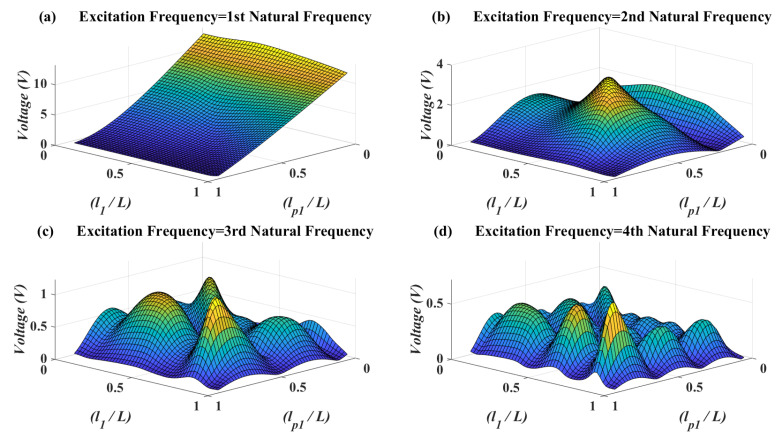
Piezoelectric voltage amplitude for different attachment location versus piezoelectric patch location—M=μL, J=0, $x_{F}=L$.

In Figure 5, the effects of patch location vs. the attachment location have been illustrated on the voltage amplitude. As can be seen in this figure, it is obvious that when the excitation frequency is near to first natural frequency, the best piezoelectric patch location and attachment location are near to the clamped end. The reason for piezoelectric patch location is that in first vibrational mode shape it has the most possible strain close to clamp side of the beam. The reason for optimum attachment location is that the force is also applying at the end of the beam and in the next figure it will become clear that having the attachment and force at the same point of the beam should be avoided. When the excitation frequency approaches to the second natural frequency, then the best placement for the piezoelectric patch and attachment is near to the midpoint of the beam. On the other hand, for the excitation frequency near to higher natural frequencies, there are many local optimums for the mass and piezoelectric patch location.

**Figure 6:**
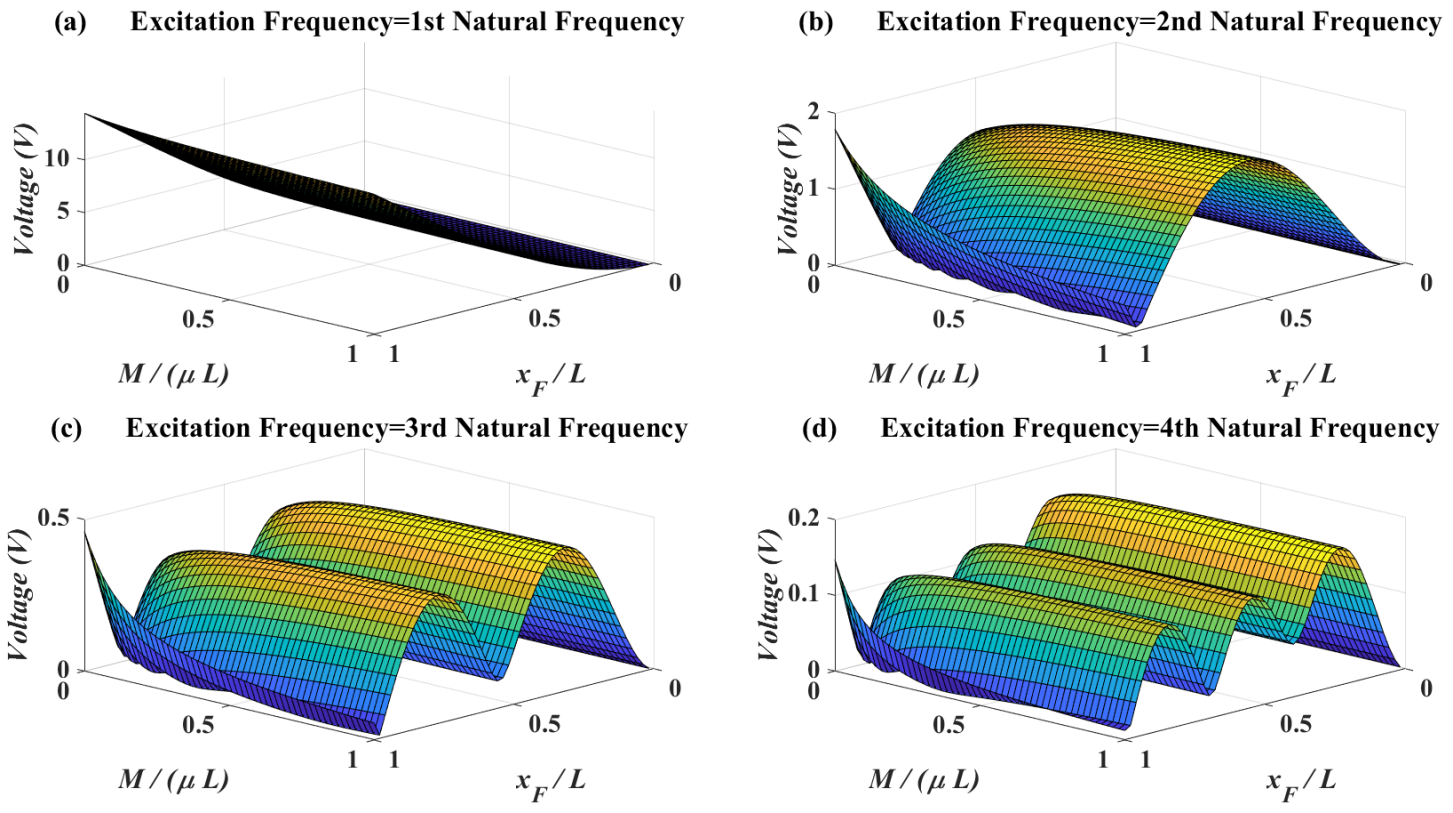
Piezoelectric voltage amplitude for different attachment mass vs. force location—$l_{1}=L$, J=0, $l_{p1}=0.01L$.

In Figure 6, the effects of attachment mass vs. force location on the voltage amplitudes are shown. Based on this figure, attachment mass does not have optimums for voltages. It means that tuning the mass for matching the natural frequency with the excitation frequency is enough. On the other hand, for the excitation frequencies close to first natural frequencies the best force location is at the tip of the beam while for excitation frequencies close to the higher natural frequencies the optimum force location is close to the mode shape apexes. Another important point is that for the excitation frequencies close to higher natural frequencies, when the force and attachment are at the tip of the beam, increasing the mass decreases the voltage amplitude. As such, when the attachment is at the tip of the beam the optimum location for force is not at the tip of the beam.

**Figure 7:**
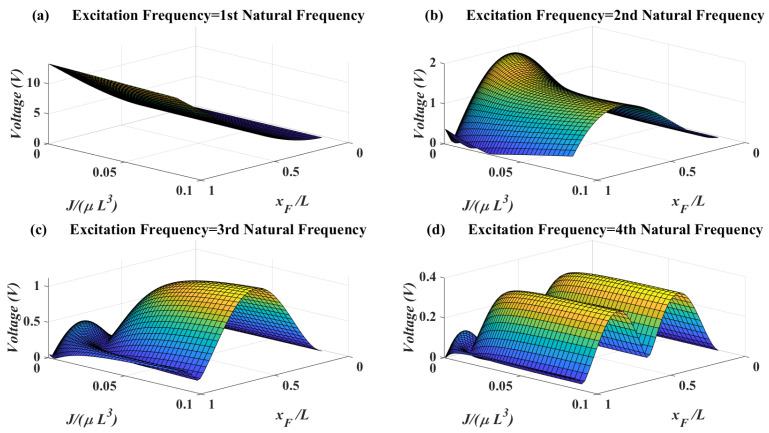
Piezoelectric voltage amplitude for different attachment mass moment of inertia vs. force location—$l_{1}=L$, M=μL, $l_{p1}=0.01L$.

In Figure 7, the effects of attachment mass moment of inertia and force location on the voltage amplitude are shown. When the excitation frequency is equal to the first natural frequency, there is no optimum for the mass moment of inertia. When the excitation frequency reaches to second natural frequency, the behavior of voltage amplitude is highly dependent on the force location. On the contrary, for third and fourth natural frequency it can be deduced that increasing the mass moment of inertia will increase the voltage generally. It will be shown in the next section that mass moment of inertia will increase the bandwidth of accessible natural frequencies to match with the excitation frequency.

### 4.2. MLP Neural Network Fitting Function

**Figure 8:**
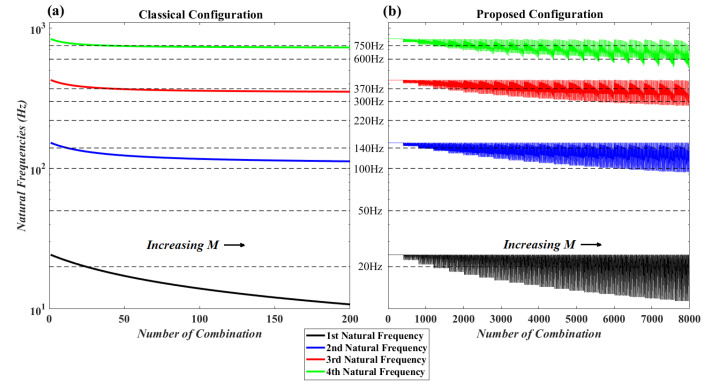
Natural frequencies for different combinations of the attachment parameters with and without considering mass moment of inertia.

In Figure 8, all possible accessible range of natural frequencies for the classical setup of cantilever with tip attachment and for the proposed setup of this paper is shown. Figure 8a illustrates the natural frequencies when the mass moment of inertia is zero and the attachment is at the tip of the beam. Figure 8b illustrates the natural frequencies for all 8000 combination of mass, mass moment of inertia and location. As can be seen, by considering in-span attachment and mass moment of inertia, a higher frequency bandwidth becomes accessible for the system of cantilever beam and attachment. For example, when excitation frequency is equal to 100 (Hz), 300 (Hz) or 600 (Hz), without considering in-span attachment and its mass moment of inertia, it is impossible to match the natural frequency of the system with the excitation frequency. On the other hand by increasing the mass moment of inertia these frequencies become accessible as it is shown in Figure 8b. Based on Figure 8, for some excitation frequencies such as 20 (Hz), 100 (Hz), 140 (Hz), 300 (Hz), 370 (Hz), 600 (Hz) and 750 (Hz), there are infinite combinations that match the natural frequencies of the system to the excitation frequencies. Now an interesting question is: which of them produce more voltage amplitude? Furthermore, some other excitation frequencies such as 50 (Hz) and 220 (Hz) are impossible to match with the natural frequencies of the system even by considering the mass moments of inertia. So, in these cases is it better to eliminate the attachment? These are the questions which will be addressed in the optimization section.

### 4.3. Optimization

**Figure 9:**
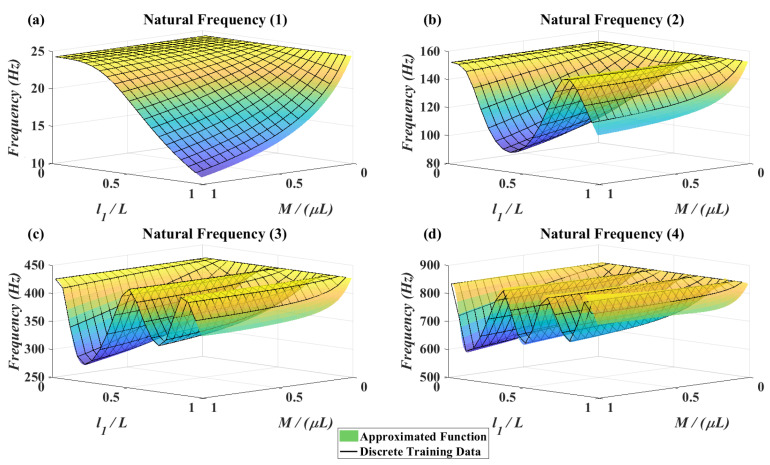
MLP neural network approximated function and discrete training data—J=0.

The trained MLP neural network is a continuous function which gets 3 inputs and gives 4 outputs. Therefore, plotting this network based on the inputs and outputs in a 3D plot is impossible. But, the mean squared error after 1000 training epochs reached to $10^{0}$ which is completely satisfying since the scale of the natural frequencies as the target data are close to $10^{2}$ (rad/s) for the first natural frequency and $5\times 10^{3}$ (rad/s) for the fourth natural frequency. To check the fitting performance of the network, it is possible to consider one of the inputs constant and plot the results for variations of the two other inputs. This is done in Figure 9, in which the four natural frequencies of the beam are illustrated based on the variation of the attachment location and attachment mass. It can be seen that the data obtained from the trained network fits the discrete training data with high accuracy. The discrete data plots for the first and second natural frequency are similar to plots obtained by Low [29].

**Figure 10:**
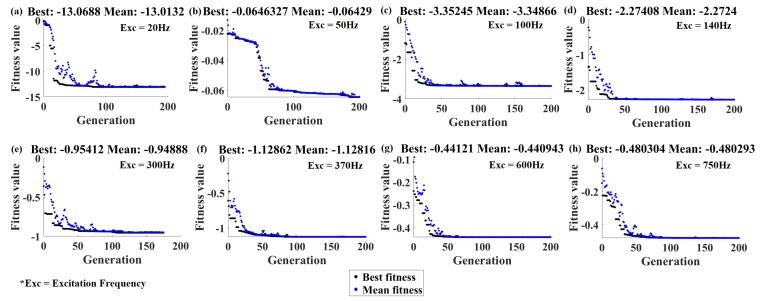
Best and mean fitness data in the generation of the genetic algorithm.

With a combination of an MLP neural network and genetic algorithm, now we have an optimization algorithm which gets the excitation frequency and gives the optimum possible optimization variables to maximize the voltage of the piezoelectric patch due to external excitation. Now, by setting the desired excitation frequency, NN based GA will choose the optimization variables based on the properties defined in Table 1. For each frequency specified in Figure 9, the best and mean fitness values in the generations are illustrated in Figure 10. It should be noted that the fitness values are obtained in the function evaluation step of GA for the output voltage multiplied to a negative sign to convert the maximization to a minimization problem. Based on Figure 10, it is obvious that less than 200 generations was enough for convergence.

In Table 2, for different excitation frequency, the optimization variables which have been chosen by NN based GA are reported. Each optimized set of variables named as config (excitation frequency).

In Table 3, the natural frequencies for optimized structures are calculated by analytical method and FEM modal analysis by COMSOL. To model the beam and in-span attachment, 3D environment of COMSOL is used. It should be noted that the cubic attachment and beam should not be in complete contact. Otherwise, the continuity of the beam will be affected by the rigidity and thickness of the attachment. Whereas, in continuity Equation (9), only the attachment mass and mass moment of inertia is taking in to consideration. To remedy a very small rigid cylindrical connector is modelled in COMSOL to mount the attachment on the beam. Furthermore, the same modal damping of analytical modelling is chosen in the COMSOL 3D environment.

**Figure 11:**
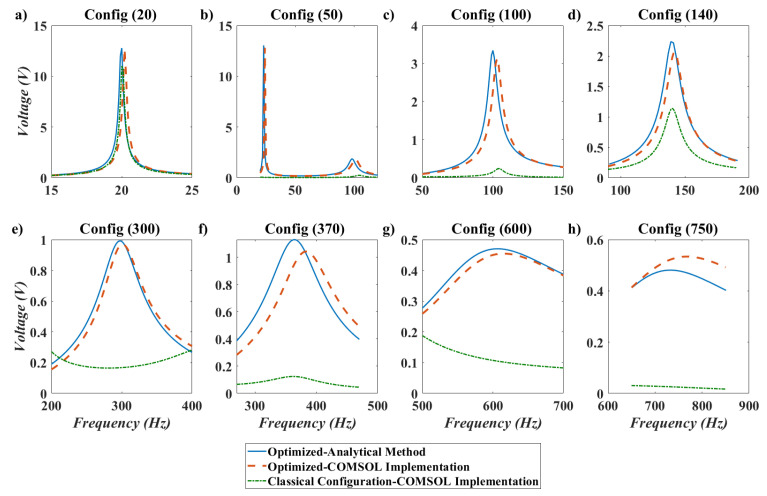
Frequency response for different configurations mentioned in Table 2.

As can be seen in Table 3, the optimization method successfully matched one of the natural frequencies of the structure with the excitation frequency, when the excitation frequency is in the accessible frequency bandwidth of the structure. It is worthwhile to mention that when the natural frequencies of the structure are calculated by analytical Euler–Bernoulli beam theory, one of the natural frequencies of the structure is exactly matches the excitation frequency with $10^{-1}$ percent error. However, when the natural frequencies of the structures are calculated by the FEM analysis, the error will be increased to $10^{0}$ percent. This increase in error comes from the fact that in 3D FEM analysis of the beam specially with attachment, there are other degrees of freedom like chord wise bending or torsional degree of freedom that are coupled with the transverse bending of the structure. This will deviate the natural frequencies of the beam in 3D FEM analysis from those of Euler–Bernoulli beam theory. To compare the piezoelectric voltages of the optimized design and classical configuration, the frequency response plot for each configuration around its excitation frequency are illustrated in Figure 11.

Several points can be deduced by the frequency response plot in Figure 11. First, it is obvious that the piezoelectric voltages derived by analytical method and FEM analysis are in good agreement. In fact, in COMSOL the Rayleigh damping is defined with same coefficient which is very important in terms of verification of the results. As can be seen in the figure, the amplitude of the voltage even at the peaks are in good agreement with the analytical calculations.

The next important point that can be seen in the frequency response plot is that, the voltage of the optimized design is highly superior to the classical configuration even for the cases when the excitation frequency is in the accessible bandwidth of the classical configuration(like 140 HZ, 370 HZ and 750 HZ). For other frequencies like 100 Hz, 300 Hz and 600 Hz which is outside the accessible bandwidth of the classical configuration, obtained voltages for optimized configurations are extremely superior to the classical configuration thanks to consideration of in-span attachment and its mass moment of inertia, which can convert the excitation frequency to resonance frequency.

## 5. Discussion

By using the evolutionary optimization algorithms like GA, it is always possible to be in the local optimums. To reduce the possibility of local optimums, GA can be applied several times with different parameters (population of individuals, mutation rate, etc.). In general, this procedure is very time consuming. However, by introducing the MPL in the core of evaluation process, the GA is performing fast enough to be applied several times with different parameters. On the other hand, even without applying the GA for several times, still the improvement of obtained results in comparison to the classical approach of tip attached cantilever beam is significant.

The optimization methodology is applied on a particular case of a beam with one in-span attachment. However, with the proposed analytical approach for the calculation of the piezoelectric voltage, the optimization methodology can be applied on a beam with several in-span attachment as well. On the other hand in this case more training data will be required for the MLP neural network.

Finally, while the optimization methodology proposed in this paper is for piezoelectric energy harvesting scope, it could be worth to explore its application to piezoelectric sensing scope. Most of the existing miniaturized piezoelectric sensors studies are focused on on fabrication technology and on their integration, but also on their combination with piezoelectric actuation in order to form one single and same structure for both actuation-sensing (named as self-sensing) [39,40,41]. Perspective works could therefore explore the use of optimization methodologies, among which the one in this paper, to design sensing or self-sensing structures with piezoelectric elements.

## 6. Conclusions

In this paper, a neural network based genetic algorithm has been proposed to maximize the voltage amplitude of a piezoelectric patch mounted on a cantilever beam with in-span attachment. The effects of attachment location, attachment mass, attachment mass moment of inertia, piezoelectric patch location and force location on the beam have been illustrated on the piezoelectric voltage amplitude. It has been shown that when the excitation frequency of a beam exceed the fundamental natural frequency, conventional configuration of cantilever beam with tip attachment and clamp side mounted piezoelectric patch does not provide the optimum voltage amplitude. The optimization results demonstrate that for excitation frequencies higher than fundamental natural frequency the piezoelectric voltage amplitude with optimization variables suggested by the proposed NN-GA has significant superiority over the conventional configuration.

Future works would extend the proposed approach to 2D structures like plates or circular diaphragms to optimize the harvested energy from the harmonic pressure. The work will also focus on the fabrication and realization of the optimized structures. Future works also include the fabrication of one optimized design from the proposed technique and perform extensive experimental tests on it. 

## Figures and Tables

**Table 1:** Vibration system properties.

Beam		Attachment		Piezoelectric Patch (PZT-4)	
$h_{b}$	0.002 (m)	*M*	$0<M<M_{b}$	$h_{p}$	0.0002 (m)
$w_{b}$	0.02 (m)	$H_{M}$	$0<H_{M}<L_{b}/10$	$w_{p}$	0.02 (m)
$L_{b}$	0.3 (m)	$W_{M}$	Varies	$L_{p}$	0.03 (m)
*E*	107 (GPa)	$L_{M}$	L/10	$C_{11}$	138.9 (Gpa)
$\rho_{b}$	2330 (kg/m${}^{3}$)	$\rho_{M}$	2330 (kg/m${}^{3}$)	$\rho_{P}$	7500 (kg/m${}^{3}$)
$M_{b}$	0.0280 (kg)	ζ	0.01	$d_{31}$	−123 (pm/V)
α	$10^{-4}$			$\varepsilon_{33}^{s}$	663.2 $\varepsilon_{0}$
β	$10^{-4}$			R	$10^{3}$ (Ohm)

**Table 2:** Optimization Variables for different excitation frequency.

		Optimization Variables				
	Excitation Frequency (Hz)	M/μL	$l_{1}/L$	$l_{p}/L$	$x_{F}/L$	$J/\mu L^{3}$
Config (20)	20	0.7940	0.5350	0.0333	1	0.0020
Config (50)	50	1	0.3310	0.0333	1	0.0010
Config (100)	100	1	0.5180	0.4640	1	0.0021
Config (140)	140	1	0.7150	0.5443	1	0.0010
Config (220)	220	1	0.2220	0.1923	1	0.0042
Config (300)	300	1	0.2957	0.6103	1	0.0010
Config (370)	370	0.9835	0.8067	0.7070	1	0.0010
Config (600)	600	0.9335	0.1547	0.7050	1	0.0034
Config (750)	750	0.4471	0.5820	0.7340	1	0.0004

**Table 3:** Natural Frequencies for different optimized configurations.

			Natural Frequencies			
	Excitation Frequency	Solving Method	1st NF	2nd NF	3rd NF	4th NF
Config (20)	20	Analytical	19.994	99.174	374.013	660.643
		COMSOL	20.21	104.67	365.99	641.64
Config (50)	50	Analytical	23.042	98.317	316.726	727.901
		COMSOL	24.12	101.32	323.79	747.15
Config (100)	100	Analytical	19.532	99.963	372.406	658.138
		COMSOL	19.519	102.88	371.99	665.84
Config (140)	140	Analytical	15.358	140.134	332.883	720.988
		COMSOL	15.339	140.81	334.15	683.85
Config (220)	220	Analytical	23.980	118.376	281.125	558.614
		COMSOL	24.48	119.12	288.56	525.31
Config (300)	300	Analytical	23.456	103.073	300.275	710.986
		COMSOL	23.154	104.24	299	690.96
Config (370)	370	Analytical	13.748	146.678	370.493	689.775
		COMSOL	13.657	147.95	379.23	689.42
Config (600)	600	Analytical	16.6254	126.09	337.366	600.502
		COMSOL	16.407	127.07	343.82	582.71
Config (750)	750	Analytical	20.9287	123.002	392.883	749.025
		COMSOL	20.515	123.55	394.62	730.83

## Appendix A. Analytical Expression for Mode Shape Constants

The constant of the mode shapes in Equation (40) are found by applying equations in (41) and the diagonal mass matrix in (43) is found by (18), all with the code written in MAPLE software as shown below,

$$\begin{array}{cc} \varphi_{1} & :=x\mapsto A_{1}\left(\sin\left(\beta \;x\right)-\sinh\left(\beta \;x\right)\right)+B_{1}\left(\cos\left(\beta \;x\right)-\cosh\left(\beta \;x\right)\right):\\ \varphi_{2} & :=x\mapsto A_{2}\left(\sin\left(\beta \;x\right)+\sinh\left(\beta \;x\right)\right)+B_{2}\left(\cos\left(\beta \;x\right)+\cosh\left(\beta \;x\right)\right):\\ \varphi_{d1} & :=x\mapsto \frac{d}{dx}\varphi_{1}\left(x\right):\\ \varphi_{d2} & :=x\mapsto \frac{d}{dx}\varphi_{2}\left(x\right):\\ \varphi_{dd1} & :=x\mapsto \frac{d^{2}}{dx^{2}}\varphi_{1}\left(x\right):\\ \varphi_{dd2} & :=x\mapsto \frac{d^{2}}{dx^{2}}\varphi_{2}\left(x\right):\\ B_{2} & :=1:\\ Constants & :=solve(\left\{\varphi_{1}\left(l_{1}\right)-\varphi_{2}\left(\tilde{l_{2}}\right)=0,\varphi_{d1}\left(l_{1}\right)-\varphi_{d2}\left(\tilde{l_{2}}\right)=0,EI\varphi_{dd1}\left(l_{1}\right)-EI\varphi_{dd2}\left(\tilde{l_{2}}\right)+\right.\\ \left.\omega^{2}J\varphi_{d1}\left(l_{1}\right)=0\right\},\left\{A1,A2,B1\right\})\\ {\bar{M}}_{rr} & :=\int_{0}^{l_{1}}\;{\left(\varphi_{1}\left(x\right)\right)}^{2}dx+\int_{\tilde{l_{2}}}^{0}\;{\left(\varphi_{2}\left(x\right)\right)}^{2}dx+M{\left(\varphi_{1}\left(l_{1}\right)\right)}^{2}+J{\left(\varphi_{d1}\left(l_{1}\right)\right)}^{2} \end{array}$$
